# High-throughput transcriptome analysis reveals a developmental increase in Acvr1c which mediates epigenetic repression of the gene encoding the pubertal brake, Makorin ring finger protein 3

**DOI:** 10.1093/narmme/ugaf008

**Published:** 2025-03-25

**Authors:** Dor Shalev, Gil Golan, Lilach Pnueli, Anat Kahan, Yael Mandel-Gutfreund, Philippa Melamed

**Affiliations:** Faculty of Biology, Technion-Israel Institute of Technology, Haifa 3200003, Israel; Faculty of Biology, Technion-Israel Institute of Technology, Haifa 3200003, Israel; Faculty of Biology, Technion-Israel Institute of Technology, Haifa 3200003, Israel; Faculty of Agriculture, Hebrew University of Jerusalem, Rehovot 7610001, Israel; Faculty of Biology, Technion-Israel Institute of Technology, Haifa 3200003, Israel; Faculty of Biology, Technion-Israel Institute of Technology, Haifa 3200003, Israel

## Abstract

Makorin ring finger protein 3 (MKRN3) acts as a “pubertal brake.” MKRN3 loss-of-function mutations are the most common genetic cause of central precocious puberty, and its expression in the brain drops significantly towards puberty, yet the drivers responsible for this reduction remain unclear. We aimed here to identify factors responsible for repressing *Mkrn3* expression across the pubertal transition, initially through bioinformatic analysis of developmental RNA-seq datasets from rodent tissues. Genes whose expression correlated significantly with *Mkrn3*, and are linked to puberty and signaling, were identified. Notably, these included *Acvr1c*, whose knockout was shown previously to delay puberty in mice. Experimentally, we show that *Acvr1c* activation suppresses *Mkrn3* via Smad2/3 signaling, involving recruitment of Kap1 and repressive histone modifications. These findings provide mechanistic explanation for the reduction in Mkrn3 required for pubertal onset, while underscoring the value of integrating high-throughput gene expression analyses with experimental validation to uncover novel regulatory pathways.

## Graphical Abstract

**Graphical Abstract:**
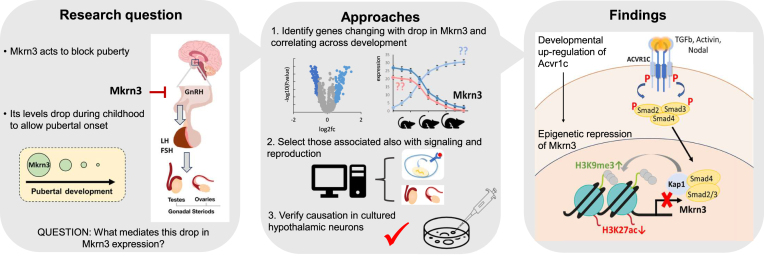

## Introduction

Central precocious puberty (CPP) is strongly associated with loss-of-function mutations in the Makorin ring finger protein 3 (*MKRN3*) gene, and these are currently the most common mutations implicated in CPP [1–4]. In the rodent and monkey hypothalamus, which comprises the control center of the reproductive axis, *MKRN3* mRNA levels decrease markedly leading up to puberty and remain low thereafter [3, 5]. These observations, together with the various roles proposed for MKRN3 in hypothalamic neurons [5–9], suggest its crucial role in inhibiting the initiation of puberty [2, 3, 10, 11]. However, the specific factors and pathways responsible for the pre-pubertal drop in MKRN3 levels have remained largely unclear.

Several studies have reported mechanisms that might regulate MKRN3 expression. One of these focused on the 3′ UTR of *Mkrn3* and demonstrated a role for mir30b whose levels increase during mouse post-natal development. This microRNA has a binding site in the 3′UTR of *Mkrn3* which, when blocked, increased Mkrn3 protein levels in hypothalamic neurons and delayed puberty in female rats [12]. Epigenetic regulation of the promoter has also been suggested to play a role, given that promoter methylation patterns were found to change across puberty in the hypothalamus of female mice [13]. In that study, 29 different transcription factors were predicted to bind the promoter, some perhaps dependent upon these changes in the DNA methylation [13].

Additional insights on possible transcriptional regulation of *MKRN3* have been gained from mutations identified in the *MKRN3* promoter that are associated with CPP. One group examined three such mutations, and suggested that two of them could lead to impaired binding of the transcription factors, HMX2 and PRDM14, while the third might create a binding site for SOX4 [14]. In a different study, a girl with CPP was found to have a 4 nt deletion in a putative binding site for the downstream responsive element antagonist modulator (DREAM) DNA-binding protein, and deletion of this element reduced reporter gene activity in a GnRH neuronal cell line [10]. Aside from these focused studies, Kap1 (KRAB-associated protein-1 or TRIM28), was found serendipitously to regulate *Mkrn3* expression levels, following a screen on the effects of its knockout (KO) in the forebrain of mice. The *Kap1* KO resulted in significant increase in *Mkrn3* mRNA levels in the hippocampus [15], though this was not the focus of that study, and its role in regulating Mkrn3 has seemingly not been studied further.

Although these findings hint at possible functional sites in the *MKRN3* promoter and various putative regulatory factors, their definitive roles in determining MKRN3 expression in the context of pre-pubertal development, and connection to pubertal timing, have not been shown. Moreover, the reduction in MKRN3 levels during late childhood is presumably driven by upstream signals and pathways that modulate transcription factor activity, so identifying these mechanisms is essential for understanding the processes that enable pubertal onset.

We sought here to identify the signaling pathways and factors that regulate *Mkrn3* expression during this pre-pubertal period, initially through bioinformatic analysis of bulk and single-cell RNA-seq datasets from brain and hypothalamic tissues across rodent development. We identified several genes whose expression is highly correlated with that of *Mkrn3*, that are related specifically to both signaling and puberty. Amongst these, genes involved in transforming growth factor beta (TGFβ) signaling pathways were notably enriched. These included *Acvr1c*, whose mRNA levels were strongly negatively correlated with those of *Mkrn3* in all datasets, and whose KO was found previously to delay pubertal onset in female mice [16]. Acvr1c is widely expressed in the hypothalamus, and our experimental work demonstrated that its activation and downstream signaling inhibits *Mkrn3* expression, involving recruitment of Kap1 and the induction of repressive histone modifications.

## Materials and methods

### Bioinformatic analysis

The RNA-seq datasets were from mice forebrain and hindrain [17], rat forebrain and hindbrain [17], and rat MBH [18], processed in R-program. Analysis of variance (ANOVA) followed by Wilcoxon signed-rank test was performed on each dataset to determine significant changes in expression at each timepoint relative to the preceding timepoint. In the bulk RNA sequencing datasets, differential expression analysis between the first timepoint that *Mkrn3* levels dropped and the one prior to it, was done with DEseq2 [19] using iDEP [20]. Pearson correlation coefficients between expression levels of *Mkrn3* and those of all other genes in the datasets were calculated based on the set of average values at each of the timepoints (*n* = 4 in all datasets) normalized and transformed (log2(CPM + 4)) counts. After combining the datasets, genes were retained if their differential expression was significant (Padj < 0.05) in all datasets, and absolute Pearson coefficient 0.6 or higher in at least 3 of the 5 datasets. All figures in this section were generated using R.

For scRNA seq data analysis, the R data serialization (RDS) and uniform manifold approximation and projection (UMAP) data files containing the Seurat object and the cell annotations created in [21] were downloaded from GEO database (GSE132355) and analyzed in R. The data were filtered initially to contain just the cells from the postnatal stages (P4, P8, P14, and P45). The filtering of hypothalamic neurons was based on the original cell annotations, and these cells filtered to contain only those that express *Mkrn3* and/or *Acvr1c*. Plots were generated using the Seurat package in R [22].

GO analysis was performed using gprofiler [23]. The selection of signaling-related genes was based on the GO database (GO:0023052). The list of genes associated with puberty was created from two datasets. The first contains genes in GWAS publications from the HuGE Navigator Gene-Phenotype Associations dataset [24], and was obtained using the search words “Puberty Precocious” from the Harmonizome 3.0 browser [25]. The second dataset was a curated list from Hou *et al.* [26]. These two datasets were merged for a collection of genes associated with puberty (Supplementary Table S1).

### Plasmid constructs

The human CA-ACVR1C (T194D) and DN-ACVR1C (K222R) pcDNA plasmids [27, 28], both gifts from Dan Bernard (McGill University, Montreal), were cut using *Nde1* and *Sac2*. The inserts were cloned into pCMV plasmid (pEGFP-C1, Clontech) cut using the same enzymes (so as not to include GFP) and verified by sequencing. Plasmids were linearized using *AseI* for stable transfections.

### Cell culture and plasmid transfections

GT1-7 mouse hypothalamic GnRH neuronal cell line was cultured, as previously [29] in high glucose Dulbecco’s modified Eagle Medium containing 10% FBS, 1% penicillin–streptomycin and sodium pyruvate (all from Biological Industries, Beit Haemek), maintained at 37°C with 5% CO_2_ at 50%–90% confluency, passaging 1–2 times a week. Cells were treated for 24 h with Activin A (5 nM, SRP6057 Sigma), Activin B (7.7 nM [100 ng/ml], 8260-AB-010/CF, R&D Systems), or SB431542 (10 μM, #13031 Cayman), doses based on published studies [30–32]. Stable transfections were carried out in cells at 50% confluency, using Lipofectamine 3000 reagent (L3000008, Invitrogen), according to the manufacturer’s instructions, with linearized plasmids, and fresh medium replaced after 24 h, followed by selection using G418 (400 μg/ml, Sigma).

### RNA extraction and real-time quantitative PCR

Total RNA was extracted using TRIzol (Ambion), treated with DNase, and purified using RNA Clean & Concentrator-5 Kit (R1014, Zymo Research). The complementary DNA (cDNA) was synthesized using qScript cDNA Synthesis Kit (Quanta Biosciences) according to the manufacturer’s instructions. Real-time quantitative PCR (qPCR) was carried out using the PerfeCTa SYBR Green FastMix (Quanta Biosciences) using primers listed in Supplementary Table S2. Amplicon levels were quantified using standard curves and normalized to the *Rplp0* housekeeping gene.

### Chromatin Immunoprecipitation

Chromatin immunoprecipitation (ChIP) experiments were carried out in formaldehyde cross-linked GT1-7 cells, as previously [29, 33–35], after extensive sonication. Antibodies (5 μl of antibody per 10 μg of chromatin) were Smad2/3 (D7G7, Cell Signaling), Kap1 (4123S, Cell Signaling), H3 (ab1791, Abcam), H3K27Ac (ab4729, Abcam), H3K9me3 (ab8898, Abcam), and IgG (ab6721, Abcam). After DNA purification, the regions of interest were amplified by qPCR (primers in Supplementary Table S2) from IP and input samples. Levels are shown as IP/input, and for histone modifications, they are normalized to H3 IP/input levels.

### Statistical analysis

The experimental data are from multiple biological repeats (*n*-value) which were assayed individually. F-test determined similarity of variance and data analyzed accordingly by Student’s *t*-test (two-tailed) to establish significance, defined as *P* < 0.05. Graphs show mean ± SEM. For multiple group testing, ANOVA followed by Tukey’s HSD post-hoc test was used.

## Results

### Analysis of RNA sequencing data identifies genes whose expression levels are strongly correlated with those of Mkrn3 across development

**Figure 1.**
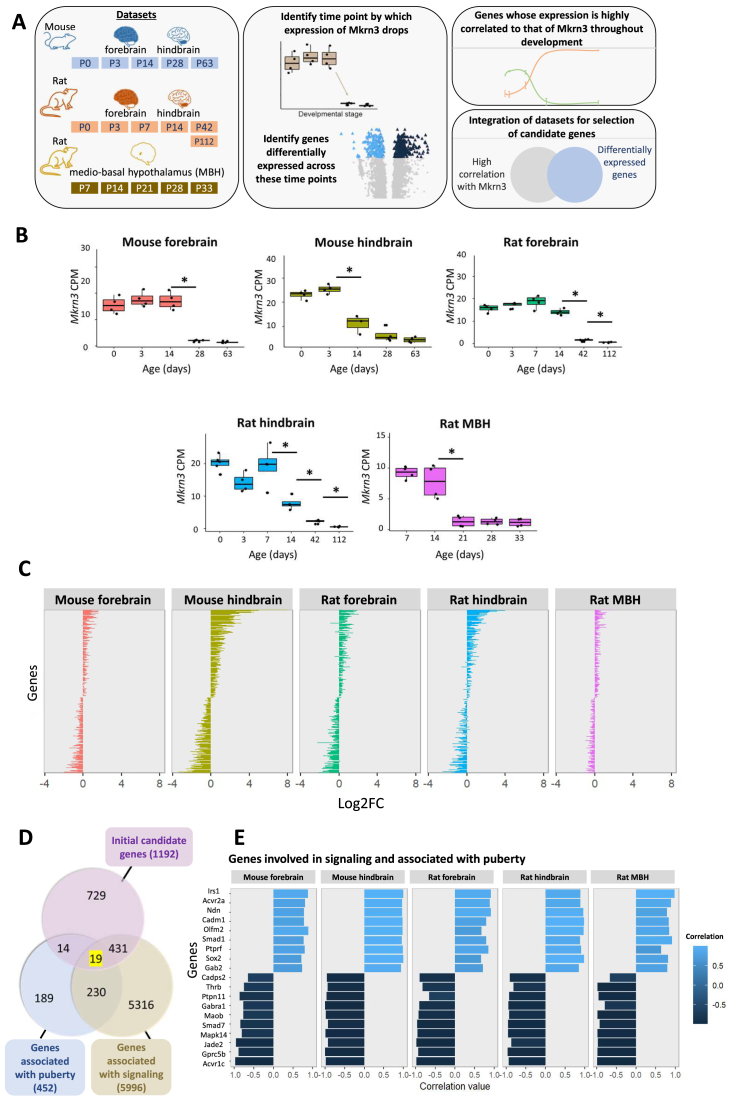
Analysis of RNA sequencing data identifies genes whose expression levels are correlated with those of *Mkrn3*across development. (**A**) Datasets and the pipeline for identifying genes whose expression is highly correlated with that of *Mkrn3*, and related to both signaling and puberty. (**B**) Boxplots show the expression levels of *Mkrn3*in the different datasets across development. Statistical analysis was conducted using ANOVA for each dataset (all *P* < 0.001), followed by Wilcoxon signed-rank tests to compare each time point with the one immediately preceding it; *P < 0.05, otherwise nonsignificant (*P* > 0.05). (**C**) The 1192 genes whose expression levels are correlated (*r* > 0.6) positively or negatively with those of *Mkrn3*in all of the datasets throughout development and are differentially expressed (Padj < 0.05) at the same time that *Mkrn3*drops in at least three of the datasets, shown as log2 of the fold-change (logFC) between the level at the time point that *Mkrn3*dropped, and at the immediately preceding timepoint. (**D**) The intersection between these 1192 genes, and genes associated with puberty and/or signaling, resulting in (**E**) 19 candidate genes (correlation value: Pearson coefficient). See also Supplementary Fig. S1.

To find genes whose expression levels are correlated with those of *Mkrn3*, we analyzed five publicly available RNA-seq datasets from forebrains and hindbrains of mice and rats, and from rat medio-basal hypothalamus (MBH) across development, including the juvenile to adulthood transition [17, 18] (Fig. 1A). We first identified the time point by which *Mkrn3* has dropped in each dataset (Fig. 1B). We then employed differential expression analysis [20] across this time point, to detect genes with changes in expression that correlate with those of *Mkrn3*. In addition, we calculated the Pearson correlation coefficient between the expression of *Mkrn3* and that of all the other genes in each of the datasets, along all time points during development. These analyses identified 1192 genes whose expression levels are correlated positively or negatively (*r* > 0.6) with those of *Mkrn3* in all of the datasets throughout development, and are differentially expressed (Padj < 0.05) at the same time that *Mkrn3* drops in at least three of the datasets (Fig. 1C). These genes are enriched for processes related to neuronal differentiation and development, as revealed by GO analysis (Supplementary Fig. S1A).

The genes arising from this screen might encode regulators of *Mkrn3*, be affected by Mkrn3, or perhaps be influenced by the same pathways, explaining their correlated expression. Given that we were looking to identify factors that regulate *Mkrn3* transcription, we focused on genes that are associated with signaling pathways, hypothesizing that changes in their activity through pre-pubertal development could regulate *Mkrn3*expression. Among the initial candidates, 450 genes are associated with the term “signaling” (Fig. 1D). We further filtered this list to pinpoint genes associated with puberty or pubertal onset. This narrowed the dataset to 19 genes whose expression levels are highly correlated with those of *Mkrn3*, related to signaling and associated with puberty (Fig. 1D and E).

Notably, 6 of these 19 genes are part of, or specifically regulated by TGFβ signaling pathways: activin receptors type 1C and 2A (*Acvr1c*, which is also known as *Alk7*, and *Acvr2a*), the downstream transcription factors *Smad1* and *Smad7*, and *Olfm2* and *Mapk14* that are regulated by TGFβ [36, 37]. *Acvr1c* expression was strongly negatively correlated to *Mkrn3* expression in all five of the datasets, and significantly increased at the same point that *Mkrn3* drops (Fig. 1E and Supplementary Fig. S1B). This was particularly striking because Acvr1c knockout in mice was shown previously to delay puberty onset [16]. Moreover, *Smad7* expression is induced by Acvr1c [38, 39] and, accordingly, its mRNA levels also increased throughout puberty in all of the datasets, in significant negative correlation with those of *Mkrn3* (Fig. 1E). These findings suggest that the increase in Acvr1c expression across pre-pubertal development might be responsible for the reduction in Mkrn3 levels that allows puberty to occur.

### Expression of Mkrn3 and Acvr1c is negatively correlated in mice hypothalamic neurons

*Mkrn3* is clearly expressed throughout the brain, and the drop in its expression across puberty was evident in all of the datasets. However, the control center of the reproductive axis resides in the hypothalamic neurons, so we went on to analyze single-cell RNA sequencing (scRNA-seq) data from the hypothalami of mice at various developmental stages [21], focusing on postnatal (PN) days 4–45, in order to identify the cell types expressing *Mkrn3*, and determine whether the negative correlation with *Acvr1c* is apparent in these cells.

**Figure 2.**
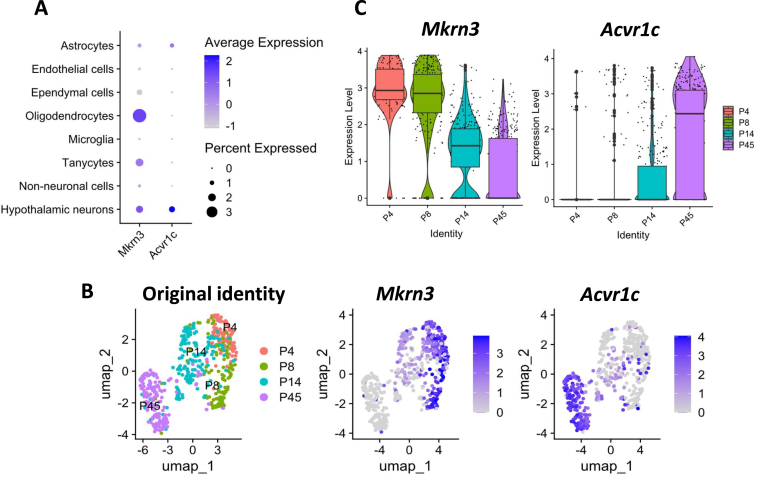
Expression of *Mkrn3*and *Acvr1c*is negatively correlated in mice hypothalamic neurons. (**A**) Dot plot showing *Mkrn3* and *Acvr1c* expression in cells from hypothalami of P4–P45 mice (data from [21]). The size of the dot represents the percentage of cells in the cell type expressing the gene, and the brightness represents the average expression level (normalized counts) across all cells in the cluster. (**B**) UMAP of hypothalamic neurons from four postnatal stages which are color-coded (P4–P45), and *Mkrn3* and *Acvr1c* expression levels (normalized counts) on this UMAP. (**C**) The normalized expression levels of *Mkrn3* and *Acvr1c* in the cells from these different age groups are shown in violin and box plots.

In the hypothalamus, *Mkrn3* was seen to be expressed most highly in oligodendrocytes, tanycytes and neurons, while *Acvr1c* is expressed in the neurons and astrocytes (Fig. 2A). Thus, the only hypothalamic cell type expressing both *Mkrn3* and *Acvr1c* are the neurons. To establish whether *Mkrn3* and *Acvr1c* are negatively correlated in these cells, we analyzed the postnatal hypothalamic neurons that express either one, or both, of these genes. Strikingly, of the 511 cells identified, only two (from PN14) expressed both *Mkrn3* and *Acvr1c* (Fig. 2B and C). These findings confirm that the dynamic and opposing changes in expression of these genes during development do occur in the hypothalamic neurons.

### The activity of Acvr1c and downstream Smad signaling pathway repress *Mkrn3*expression in a GnRH hypothalamic neuronal cell line

**Figure 3.**
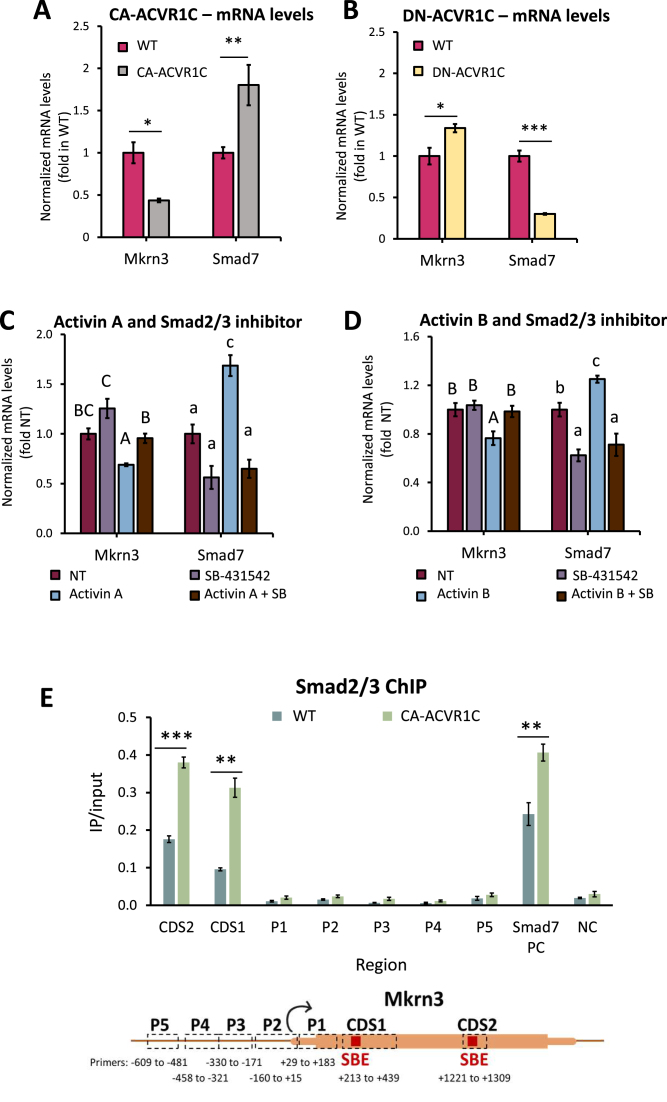
The activity of Acvr1c and downstream Smad signaling pathway repress *Mkrn3*expression in a GnRH hypothalamic neuronal cell line. (**A**) A constitutively active (CA) or (**B**) dominant negative (DN) form of the ACVR1C was stably expressed in GT1-7 cells. *Mkrn3* and *Smad7* mRNA levels were measured by qPCR and are shown after normalization to *Rplp0*, relative to levels in wild-type GT1-7 cells. Mean ± SEM, *n* = 4; Student’s *t*-test was performed between the WT and over-expressing cells. *P< 0.05, **P< 0.01, ***P< 0.001. (**C**and**D**) GT1-7 cells were treated for 24 h with (C) activin A (5 nM) or (D) activin B (7.7 nM) in the presence or absence of the inhibitor SB431542 (10 μM). The mRNA levels of *Mkrn3* and *Smad7* were assessed and are presented as before, relative to levels in non-treated (NT) controls. Mean ± SEM, *n* = 3 (in C) or 6 (in D). ANOVA followed by Tukey’s HSD post-hoc test was performed and the same letter (in upper or lower case for each gene) denotes means that are not significantly different (*P*> 0.05). (**E**) ChIP was performed for Smad2/3 in GT1-7 WT and CA-ACVR1C cells, and IP and input levels measured across the locus by qPCR, using the primer pairs shown in the schematic beneath the graph (SBE: Smad-binding element). *Smad7* serves as a positive control (PC) and ACVR1C-responsive gene; NC: negative control region. Results are shown as IP/input; mean ± SEM, *n* = 4. Student’s *t*-test compared levels between the two cell types, presented as before (Fig. 3A and B). See also Supplementary Fig. S2.

The above findings, together with previous reports of delayed puberty in *Acvr1c* KO mice [16], led us to surmise that Acvr1c-mediated pathways might inhibit *Mkrn3* expression in hypothalamic neurons. To examine this possibility, we over-expressed either a constitutively-active (CA) or dominant-negative (DN) variant of ACVR1C in the murine GnRH hypothalamic neuronal GT1-7 cell line. This resulted in a significant reduction, or increase, in *Mkrn3* mRNA levels, respectively, which contrasted very clearly with the responses observed for the *Smad7* control gene (Fig. 3A and B).

We next verified whether exposure of the cells to ACVR1C ligands also represses *Mkrn3* expression, and treated the cells with activin A or activin B. Exposure to either ligand resulted in a significant drop in *Mkrn3* mRNA levels which was abolished by addition of the ACVR1B/C inhibitor SB-431542 [30] (Fig. 3C and D), confirming that activation of this signaling pathway indeed inhibits *Mkrn3* expression.

The SMAD2 and SMAD3 transcription factors, which mediate ACVR1C signaling, have been found at the *MKRN3* promoter in other cell types (Supplementary Fig. S2). We thus performed ChIP to determine whether these factors bind the *Mkrn3* locus also in the hypothalamic neuronal cells. Smad2/3 binding was not evident at the *Mkrn3* promoter but was clearly enriched at two regions in the coding sequence, both of which contain Smad-binding element (SBE) motifs (according to JASPAR [40, 41], *P* = 10e-4). Notably, the Smad2/3 binding at both sites was significantly elevated in the cells over-expressing CA-ACVR1C, reaching similar levels to those at the positive control *Smad7* gene promoter (Fig. 3E).

### Activation of Acvr1c increases Kap1 binding and induces repressive chromatin at the *Mkrn3*locus

**Figure 4.**
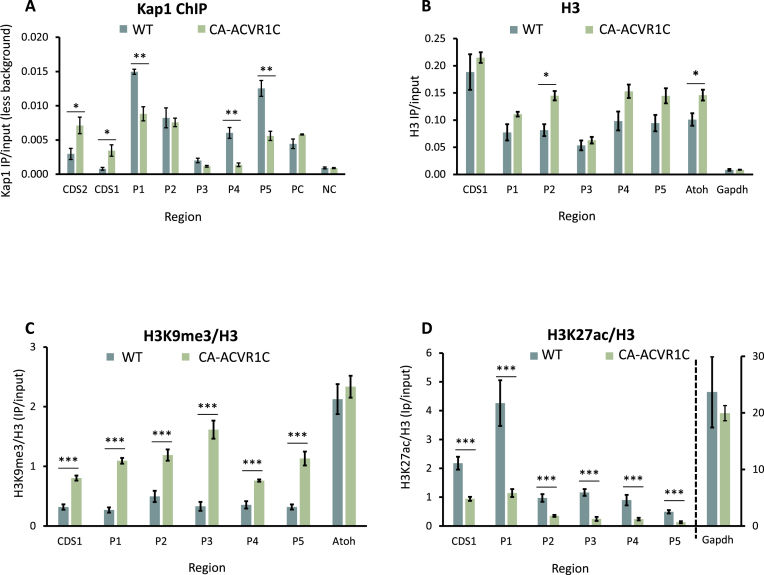
Activation of Acvr1c increases Kap1 binding and induces repressive chromatin at the *Mkrn3*locus. (**A**) ChIP was performed for Kap1 in GT1-7 WT and CA-ACVR1C cells, as in Fig. 3E, analyzed after subtraction of background (IgG) signals, and presented similarly, *n* = 3. (**B**–**D**) ChIP was performed similarly for (B) H3, (C) H3K9me3, and (D) H3K27ac. In (B), H3 values are shown as IP/input, and in (C and D), levels are shown normalized to those of H3 at the same locus. Mean ± SEM, *n* = 3. Statistical analysis is by Student’s *t*-test between the cell types, and is presented as before (*P < 0.05, **P < 0.01, ***P < 0.001). See also Supplementary Fig. S2.

Acvr1c and its activated Smad2/3 signaling thus clearly repress *Mkrn3* expression, while a previous report [15] indicated a role for Kap1 (Trim28) which was found to interact with Smad2 in a different context [42]. Moreover, Kap1 was seen to bind the *Mkrn3* proximal promoter and 5′ coding sequence (CDS; CDS1 in Fig. 3E) at some of the same sites as Smad2/3 in various cell types (ChIP-Atlas data [43] and Supplementary Fig. S2). Our own ChIP-qPCR analysis in WT GT1-7 cells revealed that Kap1 is enriched at the *Mkrn3* promoter at least to similar levels as at the positive control locus (Fig. 4A). In cells expressing CA-ACVR1C, Kap1 appeared to be recruited to the *Mkrn3*CDS, its levels increasing significantly at the Smad2/3-binding sites, while its levels at some of the promoter sites dropped (Fig. 4A).

Kap1 acts as a KRAB-scaffold protein, inducing heterochromatin via recruitment of Setdb1 which catalyzes H3K9me3, and NuRD which repositions nucleosomes and deacetylates histones [44–46]. Notably both Setdb1 and the NuRD core protein, Chd4, have been found at the *Mkrn3* promoter and 5′end of the CDS in other cell types (Supplementary Fig. S2). We therefore looked for any changes in the chromatin landscape at the *Mkrn3* locus in the CA-ACVR1C expressing cells. ChIP for H3 indicated that the promoter might be more compact in the CA-ACVR1C compared to WT cells, though the increase in H3 levels was not significant at most of the individual regions tested (Fig. 4B). However, levels of H3K9me3 were significantly increased in the CA-ACVR1C-expressing cells at all sites examined (Fig. 4C). This contrasted strongly with the levels of H3K27ac which were markedly lower at all sites (Fig. 4D), revealing ACVRC1-induced repressive epigenetic regulation of the *Mkrn3* gene.

## Discussion

Through bioinformatic analysis of developmental RNA-seq datasets spanning puberty, combined with experimental validation, we have identified Acvr1c and its downstream signaling pathway as a repressor of *Mkrn3* expression. Such a function for Acvr1c accords with previous reports that its knockout caused a delay in puberty onset in mice [16]. We now provide a mechanistic explanation for this earlier finding, given that the increase in Acvr1c expression in the brain prior to puberty explains the drop in Mkrn3 required for pubertal onset.

The reduction in *Mkrn3* levels leading up to puberty was evident in all of the datasets examined, reflecting expression levels in different parts of the brain, although effects of Mkrn3 on pubertal timing most likely occur in the hypothalamus, and have been reported specifically in Kiss1 and GnRH neurons [5, 7, 9]. The fact that *Mkrn3* and *Acvr1c* levels were negatively correlated in all of these datasets was striking and suggests a common regulatory function for this receptor. Although termed an activin receptor, like other TGFβ family receptors, ACVR1C can be activated by several different ligands, including Nodal and various growth differentiation factor (GDF) proteins [47–54], albeit with varying affinities. Several of these ligands are widely and highly expressed, playing keys roles in many aspects of development [55–57]. Such abundance of potential ligands presumably facilitates activation of this receptor in the various regions of the brain, triggering the same signaling pathway that leads to *Mkrn3* repression. However, abundant availability of potential ligands also suggests that a limited number of receptors might comprise a limiting factor, supporting the notion that an increase in expression of ACVR1C during childhood could be key in triggering the drop in MKRN3 levels.

ACVR1C signals through SMAD2/3 proteins which can stimulate or repress gene transcription as a result of their interactions with various protein partners [58–62]. Our finding that the repressive ACVR1C-induced Smad2/3 localizes to the *Mkrn3* CDS is consistent with previous observations for the position-dependent effects of transcription factors which can activate or inhibit transcription: such factors binding downstream of the transcriptional start-site (TSS) were found to be more likely repressive [63]. This Smad-mediated repression might involve various mechanisms beyond hindering progression of the polymerase, including recruitment of corepressors found to interact with these proteins in other contexts [58, 64–66]. However, previous reports that Smad2 can interact directly with Kap1 [42], and that Kap1 KO in the forebrain increased *Mkrn3* levels [15], pointed strongly to a role for this corepressor in mediating the inhibitory effects of Acvr1c on *Mkrn3*. This hypothesis was substantiated when Kap1 binding at the Smad2/3 binding sites was seen to be elevated in cells expressing the active receptor, and by the changes in H3K9me3 and H3K27ac at the locus, indicating its downstream effects on the chromatin.

These altered histone modifications can be explained by the ability of Kap1 to interact with and recruit the chromatin modifiers Setdb1 and NuRD: Setdb1 is a methyltransferase that catalyzes H3K9me3 [45, 46, 67, 68], and NuRD contains the histone deacetylases HDAC1 or HDAC2 [44, 69]. The increase in H3K9me3 and drop in H3K27ac, seen across the locus, are both associated with repressive chromatin, and likely underlie the reduction in Kap1 binding at the *Mkrn3* promoter. Kap1’s role at the promoter, which was not associated with Smad2/3, is not surprising given that it was found at this locus in other cell types, and has been shown to have both facilitating and repressive roles in transcription, via interaction with various proteins and its E3 ubiquitin and sumo ligase activity [42, 45, 70, 71]. These effects are likely position-dependent and, given the effects of its KO on *Mkrn3* expression in the brain, appear secondary to its repressive actions.

In summary, our study identifies the Acvr1c pathway as an epigenetic regulator of *Mkrn3* expression and elucidates molecular mechanisms driving changes in the chromatin landscape at the *Mkrn3* locus. This finding provides a mechanistic explanation for the reduction in Mkrn3 levels required for pubertal onset, particularly pertinent for understanding disorders of sexual maturation and pubertal timing. Furthermore, our approach of integrating high-throughput analyses of gene expression changes across development with targeted experimental validation demonstrates the power of this strategy for uncovering novel regulatory pathways.

## Supplementary Material

ugaf008_Supplemental_File

## Data Availability

Plasmids generated in this study will be distributed subject to restrictions of the original sources of their components. Datasets used are publicly available as cited in the manuscript. The code has been uploaded and is available https://doi.org/10.6084/m9.figshare.28623272.v2.
