# Supplementary material for: High-throughput transcriptome analysis reveals a developmental increase in Acvr1c which mediates epigenetic repression of the gene encoding the pubertal brake, Makorin ring finger protein 3
Source: NAR Mol Med. 2025 Mar 25;2(2):ugaf008. doi: 10.1093/narmme/ugaf008 (PMC12429960; doi:10.1093/narmme/ugaf008)
Supplement: ugaf008_Supplemental_File [file ugaf008_Supplemental_File.pdf]

SUPPLEMENTAL FILES

High-throughput transcriptome analysis reveals a developmental increase in Acvr1c which mediates epigenetic-repression of the gene encoding the pubertal brake, Makorin ring finger protein 3

Shalev at al.

Table S1: The list of puberty-associated genes

ACAD11	LIN28A	WDR25	CYP19A1	LDLR	TGFBR3
ACTB	LIN28B	WDR6	CYP1A1	LEFTY1	TH
ADARB2	LRP1B	WSCD1	CYP1A2	LEFTY2	TM7SF3
ADPRH	MAGEL2	WWP2	CYP1B1	LEPROTL1	TNF
ALOX8	MAP2K5	YY1	CYP21A2	LHB	TPH1
ARNTL	MKL2	ZFP131	CYP2A6	LHCGR	UCP1
ASCC3	MKRN3	ZFP654	CYP2B6	LIF	UCP2
BCDIN3D	NCOA7	ZKSCAN16	CYP3A4	LIPE	UCP3
BCL11A	NDN	ABCC8	DBH	MAOA	UGT1A1
BDNF	NEGR1	ACE	DDC	MAOB	UGT2B4
BEGAIN	NFAT5	ACVR1	DIAPH2	MAPK14	UGT2B7
BEND6	NPBWR1	ACVR1B	DOK5	MC2R	ZNF71
BRD8	NPHP3	ACVR1C	DOT1L	MC3R	
BRWD1	NPTXR	ACVR2A	DRD1	MC4R	
BSX	NR0B1	ACVR2B	DRD2	MCHR1	
BYSL	NR4A2	ACVRL1	DRD3	MFHAS1	
CADM1	NR5A2	ADAMTS13	DRD5	MSTN	
CADPS2	NSMF	ADAMTS9	EBF2	NBN	
CAR10	NTRK2	ADIPOQ	ECT2	NEK2	
CBX7	NUCKS1	ADRB2	EIF2B2	NEK4	
CCDC85A	ODF3	ADRB3	EIF2B4	NFKB1	
CCNL1	OLFM2	ADRBK2	EIF2B5	NFKB2	
CENPW	OLFM3	AFF2	EME1	NKX2-1	
CHD7	OLFR267	AGT	EML1	NLRP5	
COG4	OTX1	AGTR1	ESR2	NODAL	
COG6	OTX2	AGTR2	F2	NOG	
CRTC1	PAGR1A	AIRE	F5	NOS3	
CSMD1	PARP10	AKR1C2	FAM46A	NPY	
CTBP2	PCDH7	AKR1C3	FGFR1OP2	NPY1R	
DDX18	PCSK1	AKT1	FMN2	NPY5R	
DET1	PCSK2	ALDH1B1	FMO1	NR0B2	
DLGAP1	PEX2	AMH	FMO3	NR3C1	
DLK1	PGR	AMHR2	FMR1	OPRD1	
DPYD	PHF21A	ANKK1	FOXL2	OPRK1	
DST	PLCL1	ANOS1	FSHR	OPRM1	
EED	POU1F1	APOE	FST	PCM1	
EEFSEC	PRDM13	AR	GABBR2	PCSK9	
EIF4G1	PROK2	ARRB2	GABRA1	PDYN	
ESR1	PROKR2	B3GNT5	GABRB2	PENK	

ETV5	PTPRD	BCAT1	GAD2	PLIN1
EVI5L	PTPRF	BMP10	GALT	POF1B
FAM83B	PTPRK	BMP15	GDF1	POLG
FGF8	RAB7L1	BMP2	GDF10	POMC
FGFR1	RBM6	BMP3	GDF11	PPARG
FRS3	RDH8	BMP4	GDF2	PPIF
FSHB	REEP2	BMP5	GDF3	PRL
FTO	RETN	BMP6	GDF6	PRLR
FUT8	RMI1	BMP7	GDF7	PTH1R
GAB2	RORA	BMP8B	GDF9	PTPN11
GALNT10	RXRG	BMPR1A	GH1	R3HCC1
GAPDH	SATB2	BMPR1B	GHRH	ROCK1
GHR	SCRIB	BMPR2	GHRHR	SCARB1
GNPDA2	SEC16B	BUB3	GHRL	SERPINE1
GNRH1	SEC23IP	CASC1	GNB3	SHBG
GNRHR	SEMA3A	CBR3	HBE1	SLC12A7
GPR45	SEZ6L2	CCK	HIVEP1	SLC2A12
GPRC5B	SIM1	CCL5	HSD17B1	SLC2A6
GTF2I	SIRT3	CCR3	HSD17B2	SLC6A3
GTF2IRD1	SIX6	CD40LG	HSD17B3	SLC6A4
H2Q10	SLIT3	CDK12	HSD17B4	SMAD1
HACE1	SMARCAD1	CEP70	HSD3B1	SMAD2
HCRTR2	SOX10	CGA	HSD3B2	SMAD3
HDHD2	SOX2	CGB5	HTR1A	SMAD4
HESX1	STARD4	CHFR	HTR2A	SMAD5
HNF4A	STXBP4	CHRNA1	HTR2C	SMAD6
HS6ST1	TAC2	CHRNA10	HTR5A	SMAD7
HTR1F	TACR3	CHRNA2	IGF1	SORBS1
IGF2BP2	TCF7	CHRNA3	IGF1R	SPAST
IGSF11	TENM2	CHRNA4	IGF2	SRD5A1
IL20RB	TEX29	CHRNA5	IGF2R	SRD5A2
IMPG1	THRB	CHRNA6	IGFALS	SREBF2
INHBA	THRSP	CHRNA7	IGFBP1	SST
IRX3	TMEM108	CHRNA9	IGFBP2	SSTR1
JADE2	TMEM18	CHRNB1	IGFBP3	SSTR2
KCNK9	TMEM245	CHRNB2	IGFBP4	SSTR3
KCTD13	TMEM38B	CHRNB3	IGFBP5	SSTR4
KDM3B	TNNI3K	CHRNB4	IGFBP6	SSTR5
KDM4A	TRA2B	CHRND	IL1RN	STAR
KDM4C	TRAPPC9	CHRNE	IL6	SULT1A1
KISS1	TRIM66	CHRNG	INHA	TAC3
KISS1R	TRMT11	CITED4	INHBB	TACC1
KLF12	TRPC6	CNR1	INHBC	TAX1BP1
KLHDC8B	TYW3	COL1A1	INHBE	TCF7L2
LEKR1	UBA7	COMT	INS	TGFB1
LEP	VALIDPRIME	CRHR1	INSR	TGFB2
LEPR	VDR	CRHR2	INSRR	TGFB3
LGR4	VGLL3	CYP11A1	IRS1	TGFBR1
LHX3	WDR11	CYP17A1	KRAS	TGFBR2

Table S2: Primers (for ChIP unless noted otherwise)

Primer	Sequence F	Sequence R
Rplp0 (qPCR)	GCGACCTGGAAGTCCAACTA	ATCTGCTTGGAGCCCACAT
Mkrn3 (qPCR)	GCATGTGGTCTGGTCTGCTA	TTGCCTCAGTAGCCTTTGAA
Smad7 (qPCR)	CCCCCGGCTGAGAGGCTCAT	CACCTGCTGCCAGTCTGCCC
P1	GGGAACAGACACACCCTCC	CAGCTCCGCAATTCAAGCAC
P2	GACCAGCTTCTTCTCGGAG	AAGCAGAAACAGGCACGCG
P2 (histones)	GACCAGCTTCTTCTCGGAG	TGGTGTTACAACCGGAAGTG
P3	CTGGGATGGGCTTCTGCC	GCAATGTGTGTAGCGTTTTCT
P4	GCTACACACATTGCCTTTAC	GAAACGCTTTGGCAGGGAC
P5	CTGACAGGATTCTTTGGGTC	GGTCCGGGAGAATGTAACAA
CDS1	GTAGCGACAGTTATCGCCCT	AGCTTCTGCGGGAGTCAGC
CDS1 (histones)	ACTGTTGCGCTCCGGACCA	AGCTTCTGCGGGAGTCAGC
CDS2	ACTGTCTTAGGTGTATCCGC	AGAGGAGACCCTGCACTGT
Smad2/3 positive control (Smad7 promoter)	CTCTAGACCTGGGAGAGGGT	GTCTAGACACCCTGTCGCTTT
Kap1 positive control (Mest1 promoter)	GAATGTGTCCACTTAGCCAC	TGAGCCCTAAGAGTGAGTAC
Kap1 negative control (near Smad7 promoter)	GTAGCCTGAAACCACCAGAAA	CGCTGTGACCCTTTAATACAG
Smad2/3 ChIP negative control	CAGAAGTTAAGAGCACTGACTG	TAGCTTCATATGGAGGCAGG
Atoh promoter (H3K9me3 positive control)	CCCTCACTCAGGTCGCCTG	CGTGCGAGGAGCCAATCA
Gapdh promoter (H3K27Ac positive control)	GGAAGCAGCATTCAGGTCTC	CAGGATAGGACTCAGGGAATACAG

A

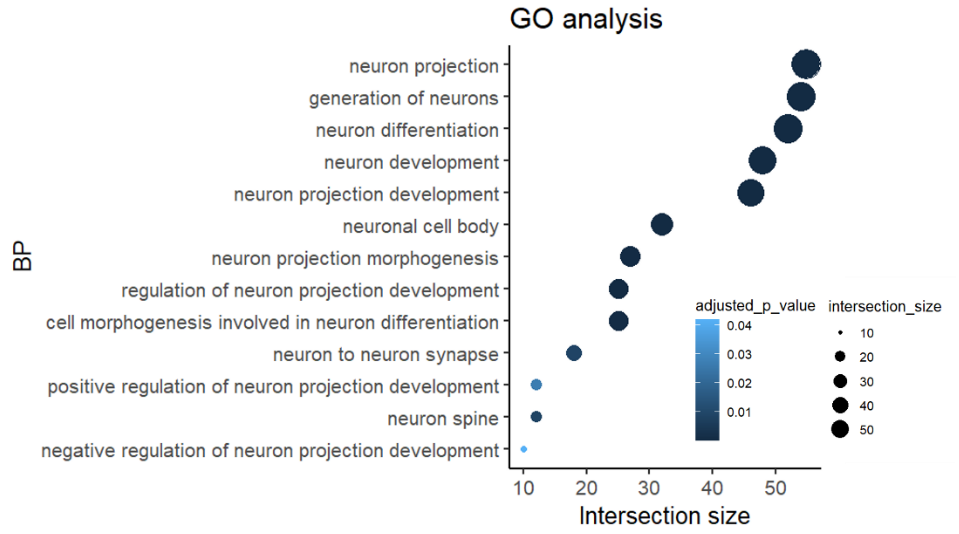

B

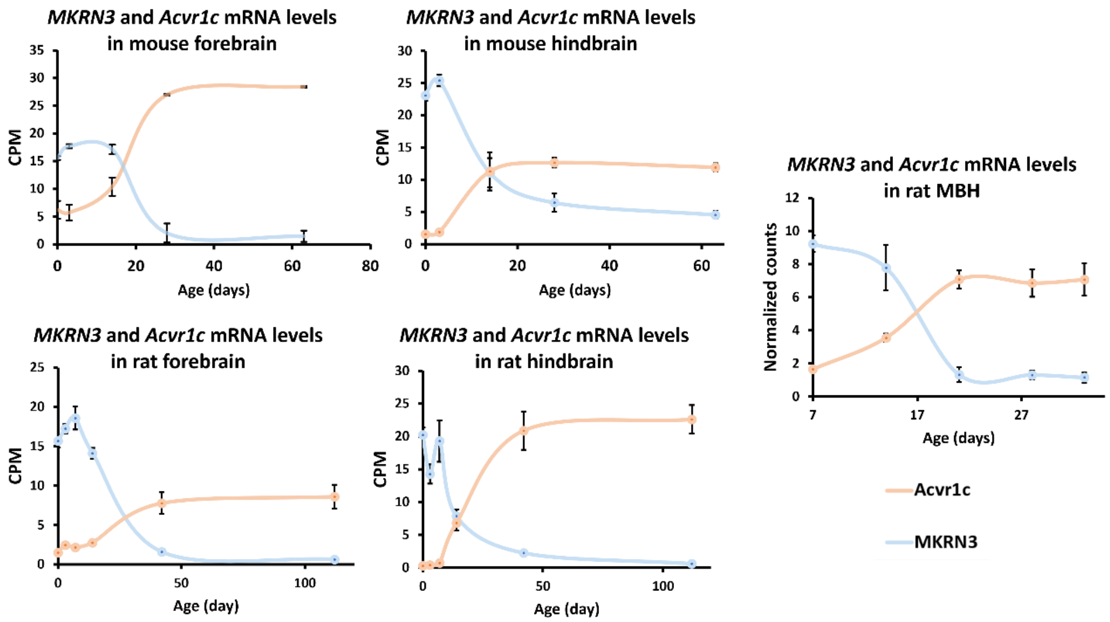

Figure S1: *Analysis of RNA sequencing data across development identifies genes whose expression correlates with that of Mkrn3*. (A) GO analysis of the enriched biological processes (BP) for the 1192 genes whose expression was found to correlate with that of *Mkrn3*. (B) Expression levels (normalized counts) of *Mkrn3* and *Acvr1c* in the various datasets across development. CPM = Counts per million.

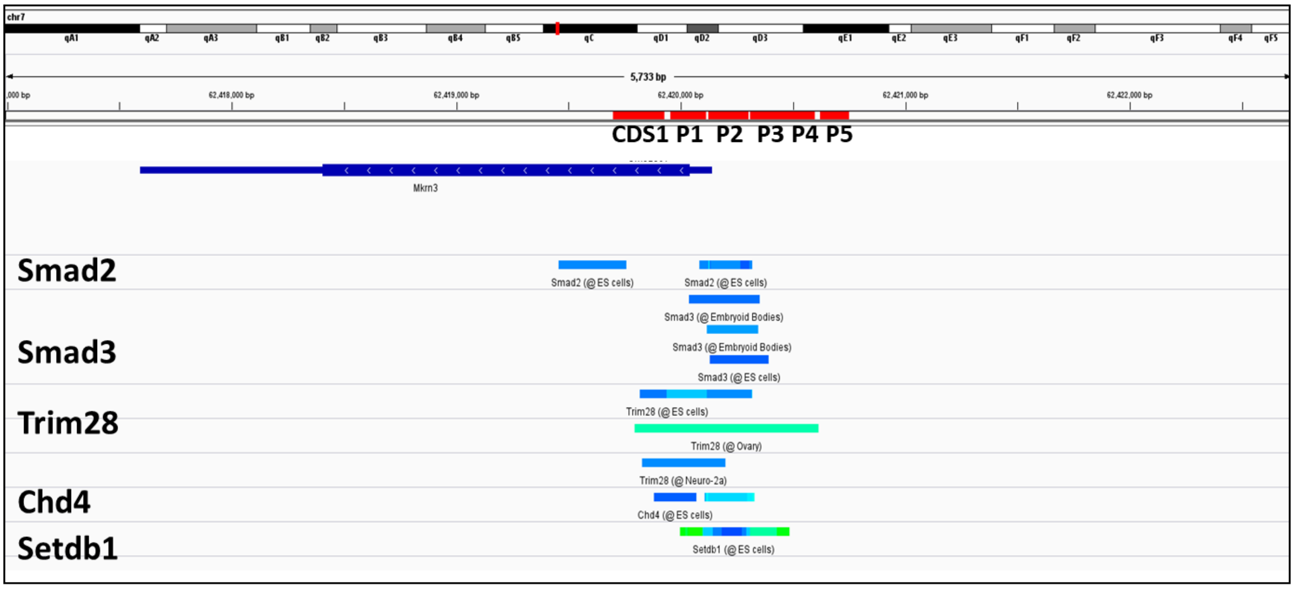

Figure S2: *Select transcription factors found previously at the Mkrn3 gene.* IGV screenshot of published ChIP data (from ChIP-Atlas) showing the *Mkrn3* gene locus and binding of Smad2, Smad3, Trim28, Chd4 and Setdb1. SBE = Smad binding element. All peaks shown are significant with p<1e-05.
